# Immune checkpoint inhibitors in advanced cholangiocarcinoma: a systematic review of efficacy, safety, and emerging biomarkers

**DOI:** 10.1186/s43046-026-00381-8

**Published:** 2026-07-06

**Authors:** Faiz Un Nisa, Moeez Ali, Talha Khan, Muskan Lohana, Aniba Asif, Nandni Kumari, Maaz Ali

**Affiliations:** 1 Isra University, Hyderābād, Pakistan; 2 International University of Kyrgyzstan, Bishkek, Kyrgyzstan; 3 Riphah International University, Rawalpindi, Pakistan; 4 University of Southern Mississippi, Hattiesburg, USA

**Keywords:** Cholangiocarcinoma, Immune checkpoint inhibitors, PD-1, Biliary tract cancer, Systematic review, Biomarkers

## Abstract

**Background:**

Cholangiocarcinoma (CCA) is an aggressive biliary tract malignancy often diagnosed at an advanced stage. For over a decade, gemcitabine-cisplatin (GemCis) remained the standard of care with limited survival benefit. The advent of immune checkpoint inhibitors (ICIs) has transformed the therapeutic landscape, but variability in outcomes and a rapidly expanding evidence base require systematic synthesis.

**Methods:**

A systematic review was conducted following PRISMA 2020 guidelines. The review was not registered in PROSPERO or another prospective registry. PubMed/MEDLINE and Embase were searched from inception through 31 December 2024. Studies evaluating PD-1/PD-L1/CTLA-4 inhibitors alone or in combination for advanced CCA were included. Risk of bias was assessed using ROBINS-I for non-randomized studies and Cochrane RoB 2 for randomized controlled trials. A narrative synthesis was performed due to clinical and methodological heterogeneity.

**Results:**

Fifty-one studies (≈ 4,800 patients) met inclusion criteria, including 8 randomized controlled trials and 43 non-randomized studies. First-line chemo-immunotherapy with durvalumab or pembrolizumab plus GemCis established new standards of care in TOPAZ-1 (mOS 12.9 vs. 11.3 months; HR 0.76) and KEYNOTE-966 (mOS 12.7 vs. 10.9 months; HR 0.83). Triplet regimens (chemotherapy + ICI + tyrosine kinase inhibitor [TKI]) demonstrated high activity (e.g., toripalimab + lenvatinib + GEMOX: ORR 80%, mOS 22.5 months). Second-line ICI monotherapy yielded modest ORRs (3–22%), while ICI + TKI combinations showed ORRs of 9–28%. Grade ≥ 3 treatment-related adverse events ranged from 10 to 17% (ICI monotherapy) to 70–75% (chemo-ICI). PD-L1 expression was not predictive in chemo-ICI; homologous recombination deficiency (HRD)/DNA damage response (DDR) mutations and circulating tumor DNA (ctDNA) clearance emerged as promising biomarkers.

**Conclusions:**

ICIs combined with chemotherapy have redefined first-line treatment for advanced CCA. Triplet and locoregional combinations show encouraging efficacy, warranting further validation. Biomarker-driven selection, particularly HRD/DDR status and ctDNA monitoring, will be critical to optimizing outcomes.

## Introduction

Cholangiocarcinoma (CCA) is a malignant neoplasm arising from the epithelial cells of the biliary tract and represents a substantial proportion of biliary tract cancers (BTCs), alongside gallbladder carcinoma and ampullary tumors [1, 2]. Based on anatomical location, CCA is categorized into intrahepatic, perihilar, and distal variants, each exhibiting different epidemiological, molecular, and clinical manifestations [2, 3]. Although uncommon, the global incidence of CCA is rising, particularly among Asian populations, and it is associated with poor outcomes due to late-stage diagnosis and aggressive biological behavior [1, 4, 5].

A significant proportion of patients present at an advanced stage, preventing curative intervention [4, 6]. For over a decade, the combination of gemcitabine and cisplatin (GemCis) has remained the standard therapy for advanced CCA; however, the outcomes remain inadequate, with only slight improvement in overall survival [3, 6, 7]. These limitations highlight the need for innovative therapeutic strategies.

In recent years, advances in cancer immunology have led to the development of immune checkpoint inhibitors (ICIs), which have significantly changed the treatment of multiple tumors [7, 8]. ICIs targeting programmed cell death protein-1 (PD-1), programmed death-ligand 1 (PD-L1), and cytotoxic T-lymphocyte-associated antigen 4 (CTLA-4) work by restoring anti-tumor immune responses [8, 9]. The rationale for using ICIs in cholangiocarcinoma comes from growing evidence of an immunosuppressive tumor microenvironment, with PD-L1 expression on tumor cells and the identification of predictive biomarkers such as microsatellite instability-high (MSI-H) and high tumor mutational burden (TMB) [9].

Several clinical trials and observational studies have evaluated ICI use, either as monotherapy or in combination with chemotherapy and targeted therapies, in advanced CCA. Some studies show promising anticancer effects and sustained responses. Overall, the effectiveness of ICIs remains variable, with inconsistent outcomes in progression-free survival (PFS) and overall survival (OS) across different studies. In addition, these therapies can lead to immune-related adverse events that necessitate close monitoring, especially in patients with pre-existing liver dysfunction.

Given the expanding body of literature and heterogeneity in clinical outcomes, a systematic synthesis of the current literature is required. Therefore, this systematic review aims to assess the effectiveness and safety of immune checkpoint inhibitors in advanced cholangiocarcinoma, with a focus on clinical outcomes including OS, PFS, objective response rates (ORR), and treatment-related adverse events. By integrating existing evidence, this study seeks to clarify the therapeutic role of ICIs and guide future research and clinical decision-making.

## Methods

This systematic review was conducted in accordance with the Preferred Reporting Items for Systematic Reviews and Meta-Analyses (PRISMA) 2020 statement. The review was not registered in PROSPERO or another prospective registry.

### Search strategy

A comprehensive literature search was performed using two electronic databases: PubMed/MEDLINE and Embase (via Ovid). The search strategy was developed in collaboration with a medical librarian and employed a combination of controlled vocabulary terms (MeSH and Emtree) and free-text keywords related to three core concepts: cholangiocarcinoma, immune checkpoint inhibitors, and clinical outcomes. Boolean operators (AND/OR) were applied to refine and optimize the search strategy. Representative search combinations included:

- (“Cholangiocarcinoma“[MeSH] OR “cholangiocarcinoma“[tiab] OR “bile duct cancer“[tiab] OR “biliary tract cancer“[tiab]) AND (“Immune Checkpoint Inhibitors“[MeSH] OR “immune checkpoint inhibitor“[tiab] OR “PD-1 inhibitor“[tiab] OR “PD-L1 inhibitor“[tiab] OR “CTLA-4 inhibitor“[tiab])
- (“pembrolizumab“[tiab] OR “nivolumab“[tiab] OR “durvalumab“[tiab] OR “atezolizumab“[tiab] OR “ipilimumab“[tiab]) AND (“cholangiocarcinoma“[tiab] OR “biliary tract neoplasms“[MeSH])
- (“programmed death ligand 1“[tiab] OR “PD-L1“[tiab]) AND (“cholangiocarcinoma“[MeSH] OR “intrahepatic cholangiocarcinoma“[tiab] OR “extrahepatic cholangiocarcinoma“[tiab])

### Additional search strategy

To ensure comprehensive coverage and mitigate potential indexing or database coverage gaps, the reference lists of all articles meeting the final eligibility criteria were manually reviewed (backward citation searching) to identify any additional relevant studies not captured through the initial electronic database search.

### Eligibility criteria

Studies were selected for inclusion if they met the following predefined criteria based on the PICOS (Population, Intervention, Comparator, Outcome, Study Design) framework.

### Inclusion criteria

- Population: Adult patients (≥ 18 years) with histologically confirmed cholangiocarcinoma (intrahepatic, extrahepatic, or perihilar), including those with advanced, unresectable, or metastatic disease.
- Intervention: Treatment with ICIs targeting PD-1, PD-L1, or CTLA-4, administered as monotherapy or in combination with other systemic therapies, locoregional therapies, or other immunotherapeutic agents.
- Study Design: All phases of prospective clinical trials (Phase I, I/II, II, and III) reporting original data on efficacy and/or safety outcomes.
- Language: English language publications.

### Exclusion criteria

- Preclinical animal studies, in vitro experiments, and xenograft models.
- Non-English language publications.
- Review articles, editorials, commentaries, letters to the editor, case reports, conference abstracts lacking full data, and study protocols without results.
- Studies in which data specific to the cholangiocarcinoma cohort could not be disaggregated from mixed BTC or other solid tumor populations.

### Study selection and screening process

All retrieved articles were imported into a systematic review management platform (Rayyan) for screening. The selection process followed a stepwise, two-stage approach. First, two independent reviewers screened titles and abstracts against the predefined eligibility criteria. Second, full texts of potentially relevant articles were retrieved and independently assessed by the same two reviewers for final inclusion. Disagreements were resolved through consensus discussion. Reasons for exclusion at the full-text review stage were documented and are reported in accordance with the PRISMA flow diagram.

### Data extraction

A standardized, pilot-tested data extraction form was used by two independent reviewers to collect relevant information from each included study. Extracted data items encompassed:

1. Study characteristics (first author, year, trial registration number, study design, phase, sample size).
2. Patient demographics and baseline disease characteristics (age, sex, ECOG performance status, tumor location, prior lines of therapy).
3. Intervention details (specific ICI agent, dosage, combination partner).
4. Efficacy outcomes (ORR, disease control rate [DCR], median PFS, median OS)
5. Safety data (any-grade and grade ≥ 3 treatment-related adverse events [TRAEs], immune-related adverse events [irAEs], discontinuation rates)

### Risk of bias assessment

The methodological quality and risk of bias of included non-randomized studies (single-arm Phase I and II trials) were assessed using the ROBINS-I tool [10]. The ROBINS-I tool evaluates bias across seven domains: confounding, participant selection, intervention classification, deviations from intended interventions, missing data, outcome measurement, and selection of the reported result. Each domain was rated as “low,” “moderate,” “serious,” “critical,” or “no information.”

For the eight randomized controlled trials, the Cochrane RoB 2 tool was used to assess risk of bias across five domains: randomization process, deviations from intended interventions, missing outcome data, measurement of the outcome, and selection of the reported result. Each domain was rated as “low risk,” “some concerns,” or “high risk.”

Two reviewers independently performed assessments for all studies, with disagreements resolved through consensus. Publication bias was not formally assessed due to the narrative synthesis design and heterogeneity of included studies.

### Data synthesis

Given the anticipated heterogeneity in study designs, patient populations, and ICI regimens, a formal quantitative meta-analysis was deemed methodologically inappropriate. Consequently, a narrative (qualitative) synthesis was performed. Study findings are organized by intervention type (monotherapy vs. combination therapy) and specific ICI agent.

### Final selection

Following removal of duplicates, title/abstract screening, and full-text eligibility assessment, a total of 51 articles met the predefined inclusion criteria. Of these, 41 contained mature efficacy and safety data and were included in the final qualitative synthesis; the remaining 10 were study protocols without results and were excluded from the efficacy and safety analyses.

## Results

### Study selection and characteristics

**Fig. 1:**
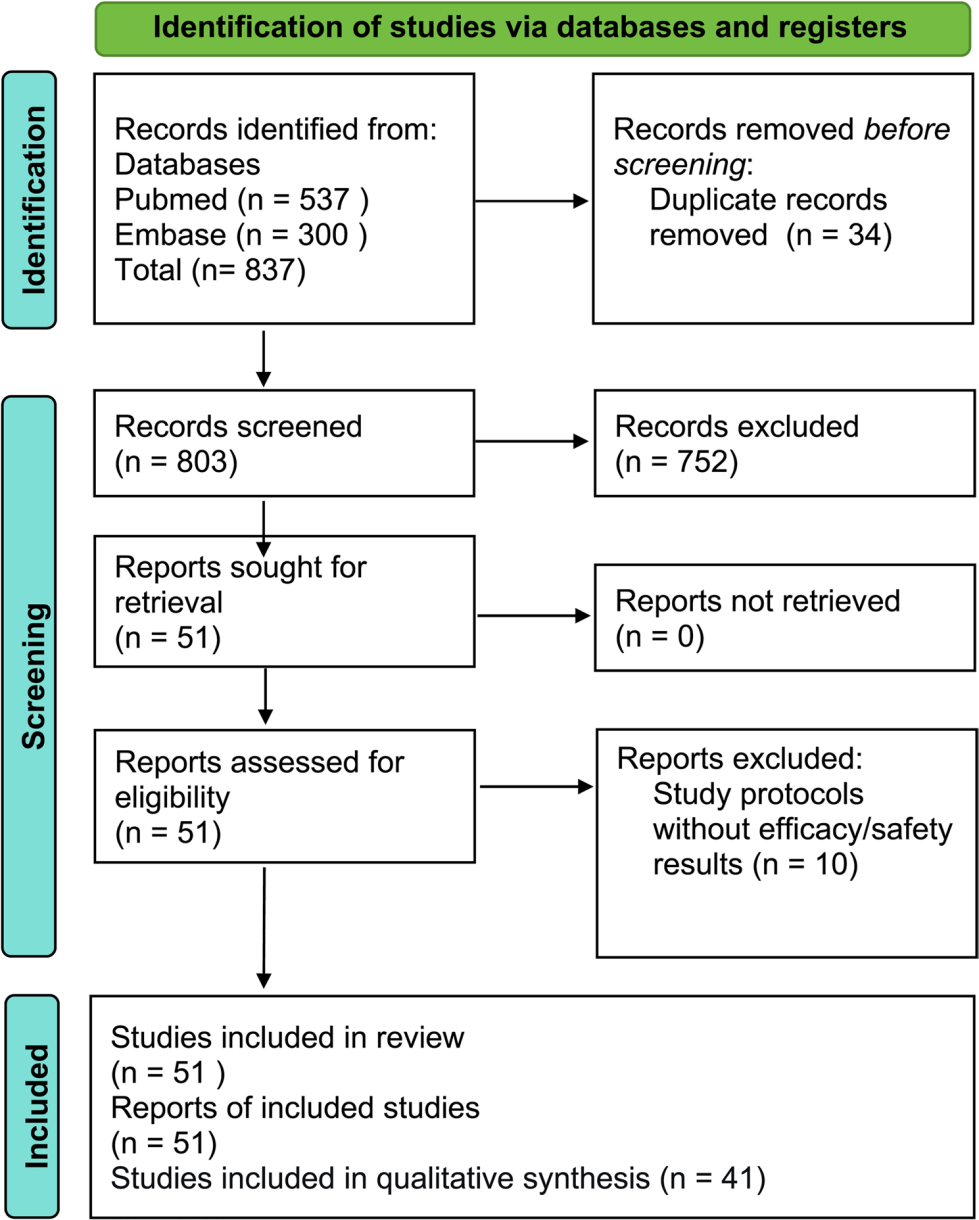
PRISMA flowchart. Legend: PRISMA: Preferred Reporting Items for Systematic Reviews and Meta-Analyses *n* = total number of records identified

The initial database search yielded 837 records (PubMed: 537; Embase: 300). From these, 51 articles met the inclusion criteria, encompassing approximately 4,800 patients across 8 randomized controlled trials (RCTs) and 43 non-randomized studies. Of these, 41 contained mature efficacy and safety data and were included in the qualitative synthesis; the remaining 10 were study protocols without results, as shown in Fig. 1.

### Risk of bias

**Fig. 2:**
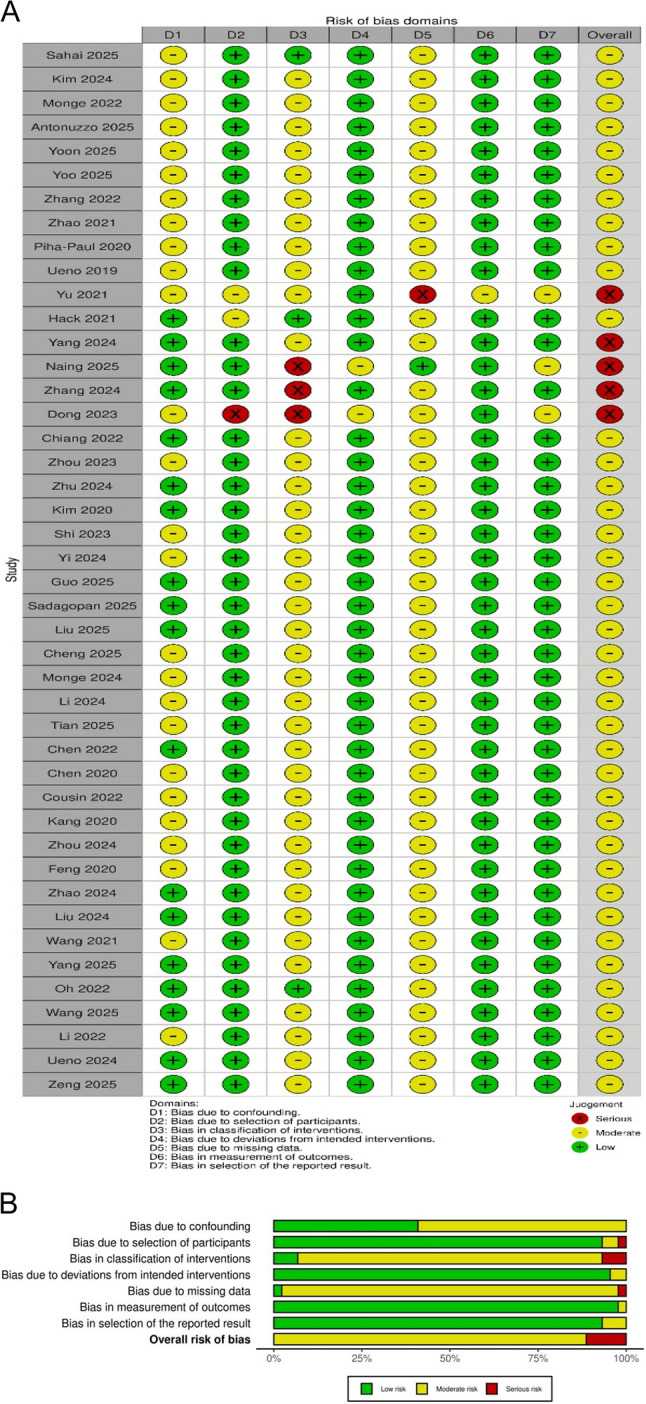
**A**: ROBINS-I, Risk of Bias Assessment tool. **B**: Cochrane RoB 2, Risk of Bias Assessment tool for randomized controlled trials (*n*=8)

Among 43 non-randomized studies assessed with ROBINS-I, 72.7% had *moderate* risk of bias, primarily due to single-arm designs (Fig. 2A*)*. The 8 RCTs demonstrated low to moderate risk (Fig. 2B).

### Efficacy outcomes

#### First-line chemo-immunotherapy

Two phase III trials established ICI plus GemCis as standard of care. TOPAZ-1 (durvalumab; *n* = 685) improved mOS to 12.9 vs. 11.3 months (HR 0.76) and doubled 24-month OS (23.6% vs. 11.5%) [1]. KEYNOTE-966 (pembrolizumab; *n* = 1,069) similarly improved mOS (12.7 vs. 10.9 months; HR 0.83) [11]. Phase II studies with nivolumab, camrelizumab, and toripalimab plus GemCis or GEMOX reported ORRs of 30.6–73.4%, supporting these findings.

#### Second-line and refractory setting

ICI monotherapy (nivolumab, pembrolizumab, toripalimab) achieved modest ORRs of 3–22% and mOS of 4.3–14.2 months, with durable responses in a subset. Chemotherapy plus ICI in the second-line (nab-paclitaxel + sintilimab) achieved ORR of 26.9% and mOS of 14.7 months. ICI plus TKI combinations (lenvatinib, regorafenib, apatinib, surufatinib, sitravatinib) yielded ORRs of 9–28% and mOS of 6.4–16.0 months [6, 12].

#### Novel combinations

Triplet regimens (chemotherapy + ICI + TKI) showed promising activity in small phase II trials. Toripalimab + lenvatinib + GEMOX in intrahepatic CCA achieved an ORR of 80% and mOS of 22.5 months [5]. GemCis + regorafenib + ICI achieved an ORR of 62.1% and mOS of 16.9 months [13]. Radiotherapy plus camrelizumab in locally advanced disease achieved an ORR of 61.1% and mOS of 22.0 months with minimal toxicity [14]. Atezolizumab + cobimetinib (MEK inhibitor) improved mPFS (3.65 vs. 1.87 months; HR 0.58) but not OS [15].

Treatment settings included first-line (*n* = 23), second-line or later (*n* = 17), and adjuvant (*n* = 1). Table 1 provides comprehensive study details and summarizes the pivotal phase III and key randomized phase II trials of ICIs in advanced biliary tract cancer.

**Table 1:** Pivotal phase III and key randomized phase II trials of immune checkpoint inhibitors in advanced biliary tract cancer

Trial (Author, Year)	Phase	*N*	Setting	Experimental Arm	Control Arm	ORR (Exp vs. Ctrl)	mPFS (Exp vs. Ctrl, mo)	mOS (Exp vs. Ctrl, mo)	HR for OS (95% CI)
TOPAZ-1 (Oh, 2024) [1]	III	685	1 L	Durvalumab + GemCis	Placebo + GemCis	26.7% vs. 18.7%	7.2 vs. 5.7	12.9 vs. 11.3	0.76 (0.64–0.91)
KEYNOTE-966 (Kelley, 2023) [11]	III	1069	1 L	Pembrolizumab + GemCis	Placebo + GemCis	29% vs. 29%	6.5 vs. 5.6	12.7 vs. 10.9	0.83 (0.72–0.95)
IMbrave151 (Macarulla, 2024) [16]	II	162	1 L	Atezolizumab + Bevacizumab + GemCis	Atezolizumab + Placebo + GemCis	26.6% vs. 26.5%	8.3 vs. 7.9	14.9 vs. 14.6	0.97 (0.64–1.47)
BilT-01 (Sahai, 2022) [17]	II	75	1 L	Nivolumab + GemCis	Nivolumab + Ipilimumab	22.9% vs. 3.0%	6.6 vs. 3.9	10.6 vs. 8.2	—
ZSAB-TOP (Shi, 2025) [18]	II	45	1 L	Tislelizumab + Ociperlimab + GemCis	—	51.2%	7.7	17.4	—
Huang, 2025 [19]	II	61	1 L	Toripalimab + Lenvatinib (Arm A)	GEMOX + Lenvatinib (Arm B)	32.3% vs. 40.0%	8.9 vs. 8.0	20.3 vs. 15.5	—
Yarchoan, 2021 [15]	II	77	≥ 2 L	Atezolizumab + Cobimetinib	Atezolizumab	3.3% vs. 2.8%	3.65 vs. 1.87	NS	—

*1 L* First-line, *2 L* Second-line, *GemCis* Gemcitabine/cisplatin, *GEMOX* Gemcitabine/oxaliplatin, *NS* Not significant

### Safety outcomes

- ♦ ICI Monotherapy: Grade ≥ 3 TRAEs occurred in 10–17% (fatigue, rash, hypothyroidism), with irAEs ≥ 3 in < 5%.
- ♦ Chemo-ICI: Grade ≥ 3 TRAEs were comparable to chemotherapy alone (70–75%), predominantly neutropenia, anemia, and thrombocytopenia. irAEs occurred in 14–20%, mostly grade 1–2 hypothyroidism.
- ♦ ICI + TKI: Grade ≥ 3 TRAEs ranged from 30 to 60%, with hypertension, hand-foot syndrome, and fatigue being class-specific.
- ♦ Triplet regimens: Grade ≥ 3 TRAEs ranged from 17 to 57%, driven by chemotherapy and TKI components.
- ♦ Radiotherapy + ICI: Notably low grade ≥ 3 AEs (8–14%).

Table 2 presents safety outcomes stratified by treatment class.

**Table 2:** Safety outcomes by treatment class

Treatment Class	Grade ≥ 3 TRAEs (%)	Most Common Any-Grade TRAEs	Most Common Grade ≥ 3 TRAEs	irAEs (Any Grade, %)	Notable Class-Specific AEs
ICI Monotherapy	10–17%	Fatigue, rash, pruritus, hypothyroidism	Rare (< 5%)	10–20%	Pneumonitis, colitis
Chemo-ICI (First-Line)	70–75%	Nausea, fatigue, anemia, neutropenia	Neutropenia (21–29%), anemia (13–19%), thrombocytopenia (8–18%)	14–20%	Hypothyroidism (mostly G1–2)
ICI + TKI (Chemo-Free)	30–60%	Hypertension, fatigue, diarrhea, hand-foot syndrome	Hypertension (15–60%), fatigue (8–15%)	20–50%	Hypertension, hand-foot syndrome, proteinuria
Triplet (Chemo + ICI + TKI)	17–57%	Neutropenia, leukopenia, hand-foot syndrome, fatigue	Neutropenia (40%), leukopenia (23%)	10–30%	RCCEP (camrelizumab), hand-foot syndrome
Radiotherapy + ICI	8–14%	Lymphopenia, fatigue, RCCEP	Lymphopenia (5–6%)	10–15%	RCCEP (camrelizumab)

*ICI* Immune checkpoint inhibitor, *TKI* Tyrosine kinase inhibitor, *TRAE* Treatment-related adverse event, *irAE* Immune-related adverse event, *RCCEP* Reactive cutaneous capillary endothelial proliferation

### Predictive biomarkers

PD-L1 expression was associated with improved response in ICI monotherapy but was not predictive of OS benefit in TOPAZ-1 or KEYNOTE-966 [1]. TMB-high status (rare in BTC; median 1.0–6.1 mut/Mb) correlated with longer PFS in some studies. MSI-H/dMMR was exceedingly rare (< 2–5%) but predicted durable responses when present. HRD/DDR mutations (present in 18.5–70% of patients; wide range reflects different sequencing panels and cohort sizes) were associated with improved ORR (71.4% vs. 33.3% in HRD/DDR-mutant vs. wild-type, respectively, in one triplet cohort) and longer PFS (HR 0.43, 95% CI 0.22–0.84) in ICI-TKI and triplet regimens [5, 6]. These findings are from small, non-randomized studies and lack prospective validation. Chromatin remodeling gene mutations predicted longer OS in chemo-ICI. VEGFA gene expression identified patients deriving PFS benefit from bevacizumab addition (IMbrave151; HR 0.44) [16]. ctDNA dynamics (post-treatment clearance) correlated with improved response and PFS across multiple studies. In one study, patients achieving ctDNA clearance had an ORR of 66.7% vs. 12.5% in those without clearance, with corresponding median PFS of 8.9 vs. 2.4 months (HR 0.31, 95% CI 0.14–0.68) [20].

### Overall findings

In this systematic review of 51 studies encompassing approximately 4,800 patients with advanced BTC, the addition of ICIs to standard chemotherapy has significantly improved survival outcomes in the first-line setting, as demonstrated by TOPAZ-1 and KEYNOTE-966, establishing durvalumab or pembrolizumab plus GemCis as the current standard of care. In the second-line and refractory settings, ICI monotherapy and ICI-TKI combinations demonstrate modest but durable activity, with a subset of patients achieving prolonged disease control. Emerging strategies including triplet regimens (chemotherapy + ICI + TKI), radiotherapy-ICI combinations, and dual ICI approaches (PD-1 plus CTLA-4 or TIGIT) have shown promising efficacy signals but require validation in larger randomized studies. Safety profiles are consistent with known drug class effects, with no unexpected toxicity signals. Predictive biomarkers remain an area of active investigation; HRD/DDR mutations, chromatin remodeling gene alterations, and ctDNA dynamics represent emerging biomarkers that may guide patient selection in future clinical practice.

## Discussion

The integration of ICIs into the management of advanced BTC, specifically CCA, has moved the field from a decade of stagnation into a rapid therapeutic evolution. This systematic review, encompassing 51 studies and approximately 4,800 patients, confirms a fundamental paradigm shift. Our synthesis demonstrates that the addition of PD-1/PD-L1 blockade to a cytotoxic backbone has effectively moved the survival needle beyond the 11.7-month mOS plateau established by the GemCis trial era [1, 2]. However, consistent with the complexities of immunotherapy, the effectiveness of these therapies is highly context-dependent, requiring a nuanced interpretation of efficacy across first-line, triplet-combination, and locoregional settings.

### Comparison to existing literature

Our findings align with broader oncological trends while offering specific evidence regarding CCA. For over ten years, standard therapy was limited by chemotherapy alone. This review confirms that the phase III TOPAZ-1 and KEYNOTE-966 trials have redefined the global benchmark, establishing chemo-immunotherapy as the first-line standard of care [1]. Notably, the doubling of 24-month survival rates observed in the durvalumab cohort (23.6% vs. 11.5%) suggests the emergence of a survival plateau, a hallmark of effective immunotherapy previously unattainable in BTC oncology [1]. The biological synergy observed here supports existing literature regarding chemotherapy’s ability to induce immunogenic cell death. By releasing tumor-associated antigens and pro-inflammatory cytokines, GemCis remodels the characteristically immune-cold biliary microenvironment into a hot state receptive to ICI activity [1, 2].

### Significant heterogeneity and methodological considerations

In alignment with the methodological rigor of high-impact research, we addressed potential sources of heterogeneity through a structured narrative synthesis. As noted in our methodology, a formal pooled analysis was deemed methodologically inappropriate due to the diverse clinical settings and significant variance in therapeutic arms. Our assessment using the ROBINS-I tool revealed that 72.7% of non-randomized studies carried a moderate risk of bias, primarily due to the single-arm nature of Phase II trials [4]. As demonstrated in our qualitative assessment, the type of data ranging from high-volume phase III trials to smaller cohort signals like the SNIPE study significantly influences the reported outcomes [3]. By opting for a narrative approach, we ensured that the nuances of drug-specific toxicities and biomarker variances were not lost to statistical averaging.

### Utility of ICIs in specific clinical settings

While doublet therapy is the current standard, our review highlights provocative efficacy signals from triplet regimens and locoregional integration:

#### The triplet synergy 

The study by Shi et al. involving toripalimab, lenvatinib, and GEMOX reported an unprecedented ORR of 80% and a median OS of 22.5 months [5]. This synergy is likely driven by vascular normalization via VEGFR inhibition. By pruning disorganized tumor vessels and reducing interstitial pressure, TKIs such as lenvatinib mitigate tumor hypoxia and facilitate T-cell infiltration [5, 6]. This magnitude of response substantially exceeds that observed in phase III chemo-immunotherapy doublets, underscoring the potential additive benefit of TKI-mediated modulation of the tumor microenvironment. In contrast, the SNIPE study [3] using a nivolumab–lenvatinib doublet without a chemotherapy backbone showed a comparatively modest ORR, emphasizing the critical role of the cytotoxic backbone in maximizing ICI efficacy.

#### Locoregional integration

A critical utility identified was the combination of ICIs with Hepatic Arterial Infusion Chemotherapy (HAIC). Zhao et al. and Zhang et al. demonstrated that targeted delivery maximizes intra-tumoral drug concentration. This approach achieved a median PFS of 11.9 months and led to significant surgical conversion rates (15.4%) for previously unresectable patients [7, 8].

#### Refractory setting progress

In second-line settings, Yoon et al. reported promising disease control with sitravatinib plus tislelizumab (DCR 65.1%) [6]. This suggests that targeting the myeloid-derived suppressor cell population in the stroma may overcome the resistance seen in unselected refractory populations [4].

### Emerging biomarkers and molecular monitoring

An essential subject of debate remains the role of predictive biomarkers. At present, no biomarker has been prospectively validated for routine clinical selection in BTC. Our review confirms that PD-L1 expression is an unreliable gatekeeper for survival benefit in BTC [1]. Instead, studies by Shi et al. and Yoon et al. point toward DDR mutations and HRD as superior candidates for patient selection [5, 6]. Furthermore, the clearance of ctDNA is emerging as a critical tool for molecular response assessment. Studies such as the Adebrelimab protocol [9] suggest that ctDNA clearance may offer a more rapid indicator of efficacy than conventional radiographic imaging [10].

### Limitations

The limitations of this systematic review are primarily due to the lack of long-term prospective data for newer triplet combinations. The retrospective design of several included studies carries a risk of selection bias, as ICIs might have been preferentially used in patients with higher performance status. Furthermore, the reliance on single-center, single-arm studies may lead to an overestimation of ORR compared to multi-center RCTs. Lastly, the significant heterogeneity in TKI partners and dosages complicates the identification of a single gold-standard triplet regimen at this time.

## Conclusion

In conclusion, the clinical application of ICIs has redefined the therapeutic standard for advanced CCA. While chemo-immunotherapy remains the current cornerstone of first-line treatment, triplet regimens and HAIC-based combinations represent emerging approaches that may facilitate surgical conversion and clinical benefit. Future clinical focus must prioritize precision oncology through biomarker-driven stratification, particularly focusing on DDR and HRD status. Advancing toward bispecific antibodies and standardized molecular monitoring via ctDNA will be essential to overcoming existing resistance and optimizing long-term survival in this challenging disease [10].

## Data Availability

All data are derived from previously published studies cited in the reference list. No new primary data were generated.
